# *Vaccinium uliginosum* L. (bog bilberry) and the search for its alleged toxicity: a review

**DOI:** 10.3389/ftox.2024.1358840

**Published:** 2024-01-31

**Authors:** Zuzana Vaneková, Patricia Holloway, Judith M. Rollinger

**Affiliations:** ^1^ Division of Pharmacognosy, Department of Pharmaceutical Sciences, University of Vienna, Vienna, Austria; ^2^ Institute of Agriculture, Natural Resources and Extension, University of Alaska, Fairbanks, AK, United States

**Keywords:** bog bilberry, *Vaccinium uliginosum L.*, toxicity, hallucinogenic, edible, phytochemistry

## Abstract

Bog bilberry (*Vaccinium uliginosum* L.) is a wild-growing berry native to all circumboreal regions. There is however a significant discrepancy in the uses of bog bilberry fruits around the world. There exists a strong prejudice against the use of these berries in many European countries as well as a few incidences of poisoning reported between 1906 and 1944. In Asia and North America, this fear is completely absent from the local knowledge and the bog bilberry is valued as an excellent food and medicinal plant. There has been a lack of research on the topic in the last 50 years and thus the presumed toxicity remains unproven. This review aims to gather the conflicting information from all regions where bog bilberry grows and present them in a critical way to elucidate the possible explanations for the discrepancies. There are several possible explanations for the alleged toxicity of the bog bilberry, including a fungal infection of the fruits, individual intolerance or accidental poisoning by a different plant species; the local names meaning “drunk, inebriating, vomit-inducing berry” may be related to the alcoholic drinks made from them. This review highlights the gap in knowledge and serves as a theoretical framework for future research.

## 1 Introduction

Bog bilberry (*Vaccinium uliginosum* L.) is a species of wild-growing berry native to all circumboreal regions. It prefers acidic, peaty soils in cold boreal and arctic climates, but is able to adjust to various environments. In lower latitudes of Asia and Central Europe it is considered a glacial relict, occurring only in higher altitudes. The northern limit is unknown, but it seems to lie a long way north of the Arctic Circle at about 70°N in north-western Alaska and to 78°N in western Greenland. It is a small bush with brown twigs bearing obovate to lanceolate deciduous blueish-green leaves with entire margins, max. 150 mm long; blooms in May-June with 5 mm long, pendulous, urn-shaped, white or pale pink flowers; the berries ripen in August-September; they grow to 10 mm in diameter, singular or in small clusters, have dusty blue peel and pale greenish-white pulp with several tiny seeds (Jacquemart, 1996).

In the past, some 30 taxa at various taxonomic levels have been described in this morphologically variable, diploid–polyploid complex species, including *V. occidentale*, *V. uliginosum* subsp. *pubescens*, *V. uliginosum* ssp. *microphyllum* and *V. uliginosum* ssp. *gaultherioides*., but nowadays *V. uliginosum sensu lato* is usually recognized as a single species that contains three distinct lineages with varied ploidy levels and unclear borders in nuclear and plastid DNA within each lineage; it is also not possible to fully distinguish them according to their morphological characteristics (Alsos et al., 2005; Regele et al., 2017).

Bog bilberry often co-occurs and can be confused with other blue-fruited *Vaccinium* species. In its European and Asian area of distribution, the most commonly mistaken species is the common bilberry, *Vaccinium myrtillus* L. (Vaneková and Rollinger, 2022). In North America, it can be found alongside other wild-growing species such as *V. ovalifolium* Sm., *V*. *cespitosum* Michx and *V. membranaceum* Douglas ex Torr. and it can be even confused with the cultivated high- and lowbush blueberries, some of which can be still found growing wild (such as the lowbush blueberry, *V. angustifolium* Aiton) (Vander Kloet, 2009). Bog bilberry can be easily identified by its blue-green obovate leaves with entire margins and by the berry apex with persisting, non-protruding calyx lobes. The distinguishing features of all listed species are summarized and compared in Table 1.

**TABLE 1:** A comparison of distinguishing features between *Vaccinium uliginosum* and other *Vaccinium* species commonly mistaken with the bog bilberry (Vander Kloet, 2009).

Morphological characteristic	*Vaccinium uliginosum* L.	*Vaccinium myrtillus* L.	*Vaccinium ovalifolium* Sm.	*Vaccinium cespitosum* Michx.	*Vaccinium membranaceum* Douglas ex Torr.	*Vaccinium angustifolium* Aiton
Habit	Small shrub	Small shrub	Taller, crown-forming bush	Dwarf shrub	Erect shrub	Small shrub
Twigs of the current season	Reddish brown, rarely green	Green, triangular	Green to brownish	Yellow-green to reddish brown	Yellow-green to reddish green	Green
Leaf margins	Entire	Serrate	Entire	Serrulate	Serrate	Serrate
Leaf color	Blue-green	Bright green	Pale green	Bright green	Bright green	Bright to dark green
Leaf shape	Obovate to lanceolate	Ovate to lanceolate	Ovate to elliptic	Oblanceolate	Elliptic to ovate	Elliptic
Berry apex	5 closed calyx lobes	Round	Round	Round	Round	5 protruding calyx lobes

In the past 20 years there has been an increased interest in utilizing the bog bilberry fruits as a food source, as well as a rich source of bioactive compounds (Määttä-Riihinen et al., 2005; Lätti et al., 2010; Ancillotti et al., 2016; Klavins et al., 2019) that might be used to alleviate the symptoms of various inflammatory, neurological and degenerative diseases (McGill, 2010; Pambianchi et al., 2021). At the same time, there are books and articles that warn against ingesting the berries, claiming they can have toxic or hallucinogenic effects (Rätsch, 2005). In some parts of the world, bog bilberry is regarded as an excellent food and medicinal source, the berries are picked and made into jams, jellies and beverages. In other countries, predominantly in Europe, they are avoided. Curiously, bog bilberry has various local names in different European countries, many of which relate in some way to inebriating effects, which are in the focus of this review. Other names usually relate either to the dusty blue color of the berries or to their wetland origin.

This review provides data from several regions where *V. uliginosum* grows and presents them in a comprehensive way, aiming to elucidate the discrepancy in the attitude towards the use of bog bilberries; previous attempts to solve the mystery of the bog bilberry toxicity are summarized, as well as the possible theories that may explain the phenomenon.

## 2 Materials and methods

Full-length research and review articles, *in vivo* and *in vitro* studies, dissertations, and case reports, obtained from international databases such as Google Scholar, JSTOR, ScienceDirect, Scopus, Web of Science, and PubMed, were included in this review on alleged toxicity of *V. uliginosum*. Following combinations of specific keywords related to the edibility, ethnopharmacology and biological activities of *V. uliginosum* fruits or fruit extracts were used as queries: (bog bilberry OR bog blueberry OR bog whortleberry OR *V. uliginosum*) AND (phytochemistry OR constituents OR toxic OR hallucinogenic OR edible). In order to include articles in languages other than English, all common names for *V. uliginosum* in relevant languages mentioned in Section 3 were also included in the search.

In addition, a large number of books and printed periodicals between 1772 and present were included. To this end, mainly the Elmer E. Rasmuson Library at University of Alaska, Fairbanks, was utilized, as it houses the largest collection of literature related to the Arctic. Other libraries and personal collections were also searched and utilized.

Lastly, a number of online articles is included in this review, in order to assess the general attitude of different nations towards bog bilberries.

All effort was made to track the cited information back to the primary source, in order to uncover possible mistakes in the translation or citation.

## 3 Ethnopharmacology and various mentions by land

**FIGURE 1:**
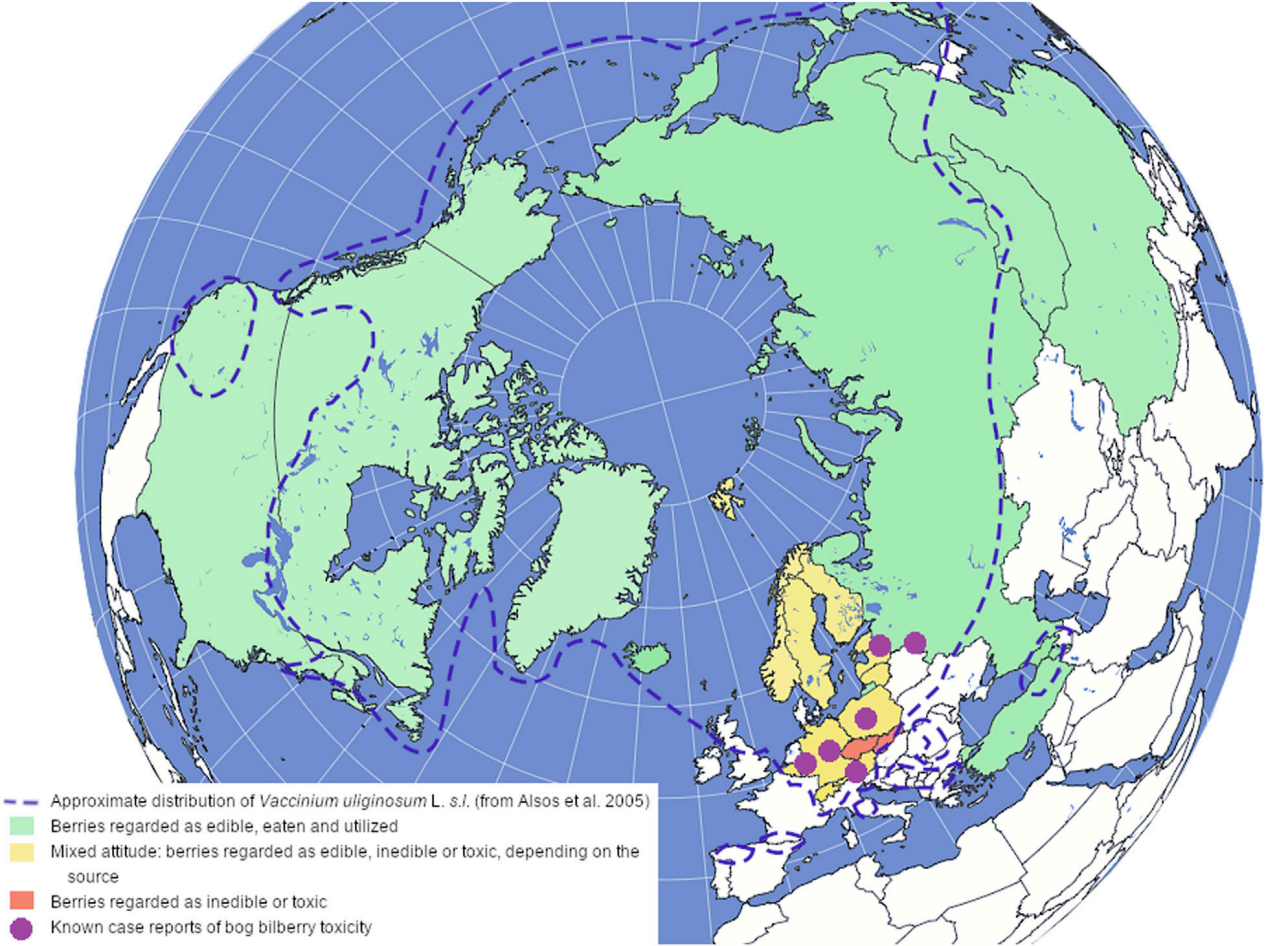
A map of approximate distribution of *Vaccinium uliginosum* L. *sensu lato* (adapted from Alsos et al., 2005), with the countries colored according to the local attitude towards the berries; the case reports of bog bilberry toxicity are described by Nevinny, 1908, Feder, 1912, Frenkiel, 1932, Kreuder, 1937, Lendle, 1943; Zipf, 1944.

In this section, the origin of the common names, ethnopharmacology, food use, case studies and various local knowledge from countries where bog bilberry grows will be reviewed. A summary of this data can be viewed in Figure 1.

### 3.1 Germany, Austria, German-speaking Europe

*Rauschbeere* (German), *Schwindelbeere* (German), both referring to “inebriating berry”; *Trunkelbeere* (German), referring to “drunken berry”.

Bog bilberry grows in vast swaths in the alpine heathlands and blanket bogs in the Alps; elsewhere in the German-speaking territory it occurs less commonly, only in raised peat bogs which, after centuries of water regulation and farming, tend to be very geographically isolated (Jacquemart, 1996).

The opinions of the local people differ significantly from each other. Some collect them along with the common bilberries (*V. myrtillus*), some refuse to eat them on the basis of being pale-colored inside, which is a differentiating property from the common bilberry (Lankmayer, 2013; Harfmann, 2018; Vaneková and Rollinger, 2022).

In 1906, the physician and pharmacologist Josef Nevinny investigated a case in Kitzbühel, Austria, when a 7-year-old girl ate bog bilberries alongside common bilberries and shortly afterwards fell ill: vomiting, diarrhea, headaches, drowsiness and general unwellness. These symptoms resolved themselves quickly without any medical intervention. Nevinny was able to identify common bilberry, as well as bog bilberry in the girl’s stool sample. He then reviewed the toxicology literature available to him at the time: Husemann and Husemann, 1862, assert that the berries possess a weak narcotic effect, but point out that they never caused any such effects to them personally. Lewin, 1897, claims that a large amount of berries causes vomiting, headaches and drowsiness, but they can be eaten in smaller amounts. Nevinny also points out that in the original works of Carl von Linné in their first German edition, a note can be found “A copious consumption of the berries supposedly intoxicates the head a little” (Linné, 1772), which was then uncritically cited by many other botanists, while others doubted the credibility of this claim (Nevinny, 1908).

In 1912, local police in Aachen, Germany, was informed about a man from Eupen (a city nowadays in east Belgium) selling „bilberries” that caused headaches to several people. These berries were confiscated and identified as bog bilberries upon inspection. This incident was described by E. Feder in the journal Pharmazeutische Zentralhalle für Deutschland. He commented on it stating: “Contrary to experiences after eating bog bilberries, not even the slightest harmful side effect has been observed when consuming common bilberries. The widespread opinion that the bog bilberries have an intoxicating effect is of course not correct. However, when consumed in large quantities, they produce headaches.” (Feder, 1912)

Probably the most famous report of bog bilberry toxicity was published in 1937 by a German medicine student, miss Friedel Kreuder: „In the middle of August 1936, I hiked with a 21-year-old friend across Wasserkuppe to Rotes Moor (a large peat bog near Fulda in Central Germany). During the hike we were looking forward to the bog bilberries (“*Rauschbeeren*”), which, as I knew from earlier hikes, grow in large quantities in Rotes Moor. I did not add any significance to the fact that they are considered poisonous or at least inedible by the local population, since I had eaten about a handful in the past year without feeling any noticeable damage. At the time, I had attributed mild fatigue and a hot feeling in my head to the hot day and the physical exertion. Around 1 p.m. we arrived thirsty and with an empty stomach to the bog and immediately fell on the big, juicy, sour berries. [...] Then we had bread, more berries for dessert, I smoked a cigarette. Overall, each of us had eaten about 250–350 g. Half an hour later we both felt as if the sun was shining on our heads. In reality it was cool, the sky covered with grey clouds. [...] We left at 2:30 p.m. Then Miss R. experienced dizziness and visual disturbances: cotton grass tufts turned blue, grass was yellow, objects more than 3 m away were blurred. Her face was pale, but she had no symptoms whatsoever from the stomach or intestines. I myself felt very well despite the hot head, was extraordinarily cheerful, laughed at Miss R., who complained of heavy feeling in her legs, and I claimed that I could jump and walk with particular ease. Miss R. could go only a few steps without a break. [...] After an hour we had reached a path on the edge of the bog. At this point, I was also overcome with a strong dizziness, visual disturbances, leaden feeling in the legs and shortness of breath with dull pain between the shoulder blades, with slight bleeding from the lip. [...] After many rest breaks, we struggled to reach the country road. [...] We stopped a car that took us back to Wasserkuppe, where we ordered strong coffee. We tried to eat chocolate, with a mild but noticeable swallowing paralysis. Our heart rate was 44/minute. Two hours later - we had to be absolutely calm—I counted 68 and 72/min., we felt refreshed, the visual disturbances had almost disappeared. […] The feeling of ants running on the tip of my nose, lips and in my right forearm persisted until late evening. When we arrived in Kleinsassen, we ate dinner with the best appetite, slept well and felt completely at ease the next day. (Kreuder, 1937, translated by Z.V.).

The German name *Rauschbeere* can, however, be also assigned to other berry species, namely, crowberry, *Empetrum nigrum* L. (Schübeler, 1873; Hartwich, 1911; Lehbert, 1913; Netoliczky, 1914). On the grounds of confusing etymology of the name *Rauschbeere*, in 1914, the German botanist Fritz Netoliczky stood strongly against the allegations of toxicity claiming that the German name *Rauschbeere* is an etymological relict, referring either to the alcoholic drinks made from them or the geographical region where the berries grow—wetlands and marshes (“*Rausch*”—cranberry bush or similar heath-like bush, “*Rusch*“—a type of wetland grass, the root word often appearing in Germanic languages with a similar meaning, likely of a Latin origin *Ruscus* (Grimm and Grimm 1893). Regarding the report of Nevinny, Netoliczky expressed the opinion that vomiting and unwellness is completely understandable when one ingests an excessive amount of tannin-rich bilberries, which may irritate the stomach (Netoliczky, 1914).

### 3.2 Slovakia, Czech Republic

*Šialenica* (Slovak), *Šálenka* (Czech)—both referring to “mad berry”; *Blevánka* (Slovak), *Blinkavka* (Czech)—both referring to “vomit berry”; *Opilka* (Slovak)—referring to “drunken berry”.

In Slovakia, bog bilberry is endangered and protected by law, as most of the former peat bog habitats have been turned into pastures and arable land. The species occurs in the peat bogs around the border with Poland, as well as in the alpine heathlands of High and Low Tatra mountains (Bertová, 1982). Despite the rarity, the plant is known as “mad berry” and generally avoided. Since bilberry picking is very popular in mountainous areas, general public is occasionally warned against the possible mix-up in the media and is told to avoid blue berries that are white inside (Plus Jeden Deň, 2016).

Bog bilberry grows in Czech Republic more commonly than in Slovakia; mainly in low elevation peaty soils, bogs and moors, as well as in heathlands of higher elevation. Bog bilberries are known as “vomit berry” and generally avoided (Hoskovec, 2007).

The mention of the supposed toxicity of the bog bilberry found its way to a popular fiction novel “Mammoth Hunters” by Eduard Štorch. In the novel, the main character, a paleolithic boy named Kopčem, was picking bilberries. After squashing one against his leg, he realized the pulp is pale-colored and stopped eating, because he remembered the advice of his elders that they could give him a headache if he ate too much (Štorch, 1918).

### 3.3 Poland

*Opilka, Pijanica* (Polish)—referring to “drunken berry”; and many others.

A thorough report of name etymology, scientific opinions and local knowledge of the putative inebriating effects of the bog bilberry centered around Poland was written in 1979 by Maria Henslowa. According to her report, local names *Pijanica*, *Opilka*, *Bujka* allude to alcohol and being drunk, whereas *Durnica* refers to intoxication and *Szalonka* to being mad or wild. The opinion of some scholars was that the symptoms of inebriation after eating more berries are just a symptom of an allergy. On the other hand, others believed that the berries contain a certain percentage of alcohol and therefore, when consumed in excess, they can give the image of intoxication (Henslowa, 1979).

**FIGURE 2:**
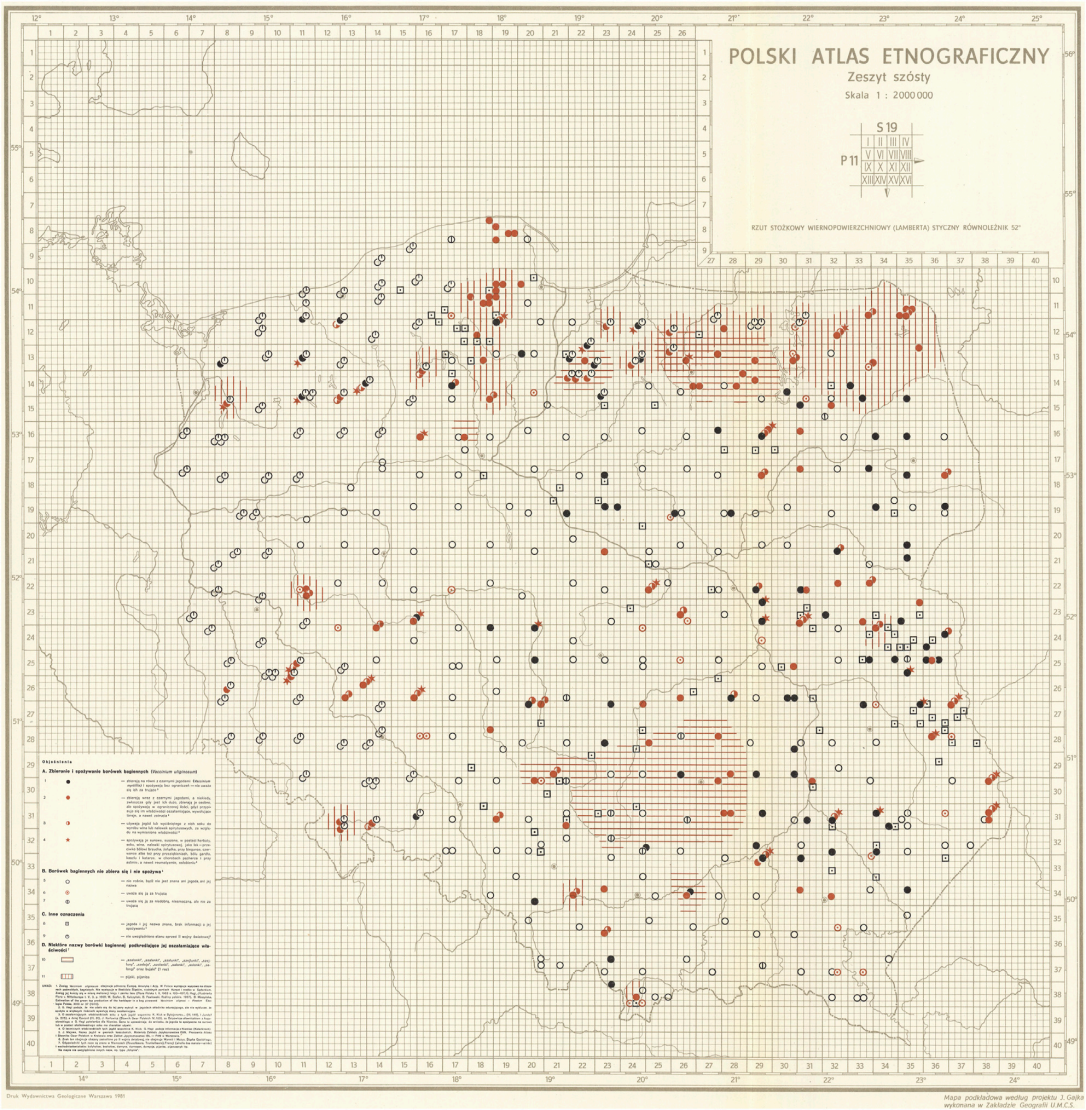
A figure from The Polish Ethnographic Atlas depicting the differing local knowledge and harvesting practices of bog bilberry fruits in Southeast Poland (Gajek, 1981, reprinted with permission from publisher. Copyright: Publishing House IAE PAN 1981). Relevant legend: Black full dots—collected, eaten and not considered poisonous; Red full circles—collected and eaten, but considered mildly poisonous; Red half-full circles—used for spirits and wine; Red stars—collected for medicinal properties; Red circles with a dot—considered poisonous; Black circles with a vertical line—considered inedible, but not poisonous; Empty black circles—plant unknown to locals; Areas marked with red lines—known as “mad” or “drunken berry”.

Data describing the differing local knowledge and harvesting practices of bog bilberry fruits in Poland was gathered in a country-wide questionnaire and published in The Polish Ethnographic Atlas (Figure 2; Gajek, 1981). The berries were reported to be widely used as a food and medicinal source by locals all over Poland wherever the species was known to them and was abundant enough to be picked. Medicinal uses included treatment of stomach aches, diarrhea, coughs and colds, bladder diseases and even rheumatism. Dietary use was reported sometimes even despite the tales of toxicity. Conflicting information could be observed in places only few kilometers apart, or even from within the same location (Gajek, 1981).

A single case report of bog bilberry toxicity exists in Poland, published in 1932 by the nurse Henryka Frenkiel from Lodz. She recalls a case of her two children, a 9-year-old daughter and a 12-year-old son, eating a handful of bog bilberries for the first time in their lives. While the girl was completely fine, the boy felt ill after 15 min, with fever, headache and nausea. Two days later the symptoms worsened with vomiting and hemolytic jaundice and the boy was transported into hospital. Jaundice soon developed into serious hemoglobinuria and anemia, worsening to the point of uncountable pulse, dyspnea and losing consciousness. In the night following day 4 the status improved sharply and unexpectedly and the boy was discharged on day 8. Chemical analysis of the berries of *V*. *uliginosum* carried out at the State Institute for Food Testing in Lodz following the incident showed that they contained a substance which had a hemolytic effect on defibrinated animal blood. On further testing, this substance proved to be a saponin-like compound. Frenkiel states that saponins are not absorbed from the stomach and gut (according to the level of knowledge in 1932), therefore it does not explain the boy’s violent reaction. Instead, she proposes a disease similar to black water fever, which is an idiosyncratic reaction of some individuals to quinin treatment of malaria, or favism, which is a reaction to beans (Frenkiel, 1932).

A unique theory exists in Poland (as well as Baltic countries, Section 3.4) that tries to explain the alleged bog bilberry toxicity: it often grows in the close proximity of another bog species, *Ledum palustre* L. (or, according to the updated taxonomy, *Rhododendron tomentosum* Harmaja). Some sources from Poland speculate that the pollen from *R. tomentosum* (which blooms in May—June) may settle on the bog bilberry plants, causing the neurological side effects, and therefore recommend a thorough washing of the fruits before consumption (Natura, 2019). This theory is not grounded in any evidence; more information to the toxicology of *R. tomentosum* can be found in Section 5.2, however no studies have been performed specifically on the pollen of this plant.

### 3.4 Baltic countries

*Joovikas* (Estonian), *Girtuoklė* (Lithuanian)—refers to “drunken berry”.

The Estonian name *Joovikas* is derived from the word *jooma* which means to drink or to be drunk. It is said to refer to the homemade wine which was made from the berries in the region. The bog bilberries are picked and eaten alongside bilberries and lingonberries. Although there exist tales of bog bilberry toxicity, the opinion of the locals is that the berries do not deserve the bad reputation. Some say the toxic effects are either a result of individual allergy or intolerance, or that they are caused by brushing against *R. tomentosum* while berry picking, which releases essential oil from the plant and intoxicates the berry picker (Kokassaar, 2006).

This was described for the first time by K. R. Kupffer in the 49th issue of Correspondence Sheet of the Natural Researchers’ Association in Riga in 1906. Firstly, Kupffer vouches for the edibility of bog bilberries with his and his colleagues’ experiences eating the berries in large amounts without any undesirable effects. He then mentions L. palustre as a new popular belief that has come up in some places but calls it an “unusual justification” and does not accept it until scientifically proven.

He then suggests two possible explanations to the differing opinions in different lands: either individual intolerance of some people, such as to strawberries, mushrooms or crab meat, or there might be a regional variance in the constituents of bog bilberries and therefore some are safe to eat and some cause headaches (Kupffer, 1906).

The previously mentioned article by Feder, published in Pharm. Feder, 1912 spurred reactions published in later volumes of the same journal. In one of these, R. Lehbert, born in the Baltic region, recalls his own experience: “On botanical excursions, I have often relied on fruits of *Vaccinium uliginosum* to quench hunger and thirst, because there was nothing else to eat, without ever suffering a headache. Only once did I feel bad: it was at the beginning of my botanical work. After several hours of marching in the Livonia—Estonian border area on a particularly hot day, I jubilantly greeted a number of very handsome specimens of “blueberries,” which I believed to be *Vaccinium uliginosum* at the time. The bushes were remarkably large, as were the juicy berries, even the leaves seemed huge. […] A twig was placed in the botanical box to show the lush fruit at home. But this did not happen immediately after returning home, as headache, dizziness and nausea soon set in, and after drinking seltzer water there was violent vomiting. «So, it is true that bog bilberries are poisonous!» was my first thought, as I started to recover. But at the same time, distrust of my own botanical competence arose, and the branch with fruit that was fortunately taken along was now “scientifically” identified, whereby it turned out that it was not *Vaccinium uliginosum* at all, but rather *Lonicera xylosteum* ^1^ (“Hundslilie, Specklilie”). This event often gave me food for thought later on; the question is justified whether what happened to a budding botanist (I was very ashamed afterwards) might not also happen to others, non-botanists, apparently confirming the assertion that the fruits of *Vaccinium uliginosum* have an emetic effect. Living on the Estonian beach 2 years ago, I often had the opportunity to eat *Vaccinium uliginosum* berries. […] Nobody suffered any ill consequences, even children who tend to be very excessive on such occasions.” (Lehbert, 1913, translated by Z.V.).

Some excerpts from older Estonian literature show a less favorable attitude towards bog bilberries in the past. In volume VI of “Eesti Entsüklopeedia” (Kleis, 1936), three types of native berries are introduced: bog bilberry, lingonberry and common bilberry; it is also added that the berries of the latter two are edible and therefore of economic importance. From this text, indirect conclusions can be easily drawn.

Similarly, in Tõnis Leisner’s review in the front issue of an Estonian science magazine Eesti Loodus in 1940, the author states that these berries are commonly on sale in Tallinn markets, but housewives often do not buy them. According to the author, people have quite different attitudes towards bog bilberries: some consider them poisonous, others not. It was often rumored that bog bilberries cause dizziness and vomiting. According to the data Leisner received from Lithuania, bog bilberries allegedly dull the senses, make the legs heavy and induce sleep. At the end of the review, the author humorously states that at least the winemakers should be happy about their possible intoxicating effect (Leisner, 1940).

### 3.5 Nordic countries

*Juolukka* (Finnish)—refers to “booze berry”; *Odon* (Swedish)—refers to “raging, wild berry”; *Fyllebär* (Swedish)—refers to “drunk berry”; *Tryte* (Norwegian) means “Whining woman”.

Norwegian names *Tryte* or *Mikkelsbær* have derogatory connotations, although derived most likely from the fact that the bog bilberry has been long regarded as an inferior, less tasty alternative to *V. myrtillus* (Furuset, 2016).

The Swedish name *Odon* consists of the nomen -*on*, which means berry, and the root word *othen* or *odher*, meaning “wild,” “crazy,” “furious“ (Söderwall, 1884). The word *Othen* is also related to the Norse god Odin, whose name stems from the meaning “the furious,” “the one who makes warriors fierce,” “lord of frenzy” or “leader of the possessed” (Bremensis et al., 1876). This is not to say that bog bilberries are directly connected to Odin or Norse warriors, but rather to demonstrate the linguistic relationship of the root word.

Contrarily, in Iceland, bog bilberry is regarded as edible and collected alongside *V. myrtillus*, eaten fresh and made into jams and liqueurs. In Icelandic climate, *V. myrtillus* tends to be rarer than bog bilberry. However, both are referred to simply as “blueberry”: *V. uliginosum* as *Bláber*, *V. myrtillus* as *Aðalbláber* (main, true blueberry) (Whitney et al., 2012; Vuijk, 2023.).

In Sami languages, bog bilberry is known as *Aettemassa*/*Ehtemas* or *Bjällo-Muörje*/*Bïenjen-muerjie*, none of which bear any negative connotations (Schübeler, 1873, www.sátni.org).

The Norwegian botanist Frederik Schübeler, when talking about the traditional uses of bog bilberries in Norway, states that the juice pressed from them was mixed with sugar and fermented to create wine in the Norwegian countryside, a practice for which is the bog bilberry much better suited than the common bilberry (he apparently tested the fermentation of both kinds himself and his findings align well with the traditional uses of Alaska native tribes, Section 3.6) (Schübeler, 1873).

Ragnar Berg from Sweden, in a reaction to the story published in Pharm. Zentralhalle 1912 (Feder, 1912, Section 3.1) replied in the later issue of the same journal that bog bilberries are picked, eaten and beloved by everybody in Scandinavia, eaten raw and cooked alike, without any noticeable toxic effects. He concludes that the claims of bog bilberry toxicity are old fairytales of the common folk (Berg, 1913).

Similarly, a Swedish-born internist and toxicologist Sven Moeschlin confirms: “I have eaten the bog bilberries in large amounts, by myself and with my friends, both in Sweden and in Switzerland, always without any noticeable side effects.” (Moeschlin, 1964).

### 3.6 North America

In the boreal and arctic regions of North America, the bog bilberry bushes grow in abundance and *V. uliginosum* is the most harvested wild berry species in Alaska (Marles et al., 2000; Holloway, 2006). They are also a major wild berry foraged by the native peoples of the region; this was reported not only by the various arctic expeditions (Anderson, 1939; Walker, 1984), but by the indigenous tribes themselves (Russell, 1994; Kari, 1995; Turner, 1997; Andre and Fehr, 2000; Collective of Elders, 2014; Ziegler et al., 2018; Fienup-Riordan et al., 2020). Medicinally, the berries and leaves alike have been used in form of tea against colds and coughs, to treat diarrhea and nausea, as well as topically to treat skin diseases (Marles et al., 2000; Pambianchi et al., 2021).

In a book “Plants that we eat,” a collection of foods and food-related practices of Iñupiat of Northwest Alaska, following characteristics of bog bilberries are described:

“Blueberries are one of the trickiest foods to preserve because they are quick to mold and ferment. Above all, they ferment! They are the strongest pickling agent of our natural foods in this region. Don’t underestimate the power of fermenting blueberries—a barrel of them only half full can foam up and overflow on the storage floor if not stirred down once or twice a day. Any meat or fat stored in blueberries will get pickled, flavored and brilliantly colored in a few days to a week. […] People can get bad stomachache from eating too many berries. Everyone eats berries as they pick. But watch the children so that they don’t overeat. Feed them well before you go picking and take along plenty of lunch including oil and fat foods. If the kids do complain of a stomachache, let them sip some extra seal oil” (Jones, 2010).

There are attempts to promote cultivation and large-scale harvesting of bog bilberries in Alaska as a way to develop local agriculture and food security (Knight, 2015). This is done mainly by managing and expanding wild stands of bog bilberries. Attempts to cultivate bog bilberry have already been successfully applied in Central Siberia, where at least five selections of bog bilberry have been made, and they are cultivated in rows similar to highbush blueberries (Holloway, 2006).

### 3.7 Russia, Siberia

пьяничник—*p’yanichnik*—refers to “drunkard”; дурника—*durnika*—means “fool”.

Bog bilberries are common in the Siberian taiga and tundra biotopes. They are known and used as a food source by the indigenous peoples of Siberia, for example, the Yakuts/Sakha (Borisova et al., 2018), Nenets (Källman, 2002), Yupik (Ainana and Zagrebin, 2014), Chukchi, Koryak and Kamchadal (Bogoraz, 1909), as well as the general non-indigenous population of Siberia (Shin et al., 2022). The berry picking has been (and still is) traditionally done by women and children.

According to the publications of the Jesup North Pacific Expedition (1897–1902) the Maritime Koryak and the Kamchadal sometimes distilled a kind of brandy from berries of *V*. *uliginosum*. They used for this a large iron kettle with a wooden cover pasted over with dough. An old gun-barrel served as a condenser. This method was learned from the Cossacks. The Chukchi, however, did not have enough berries for this purpose, or the skill to thus distil them (Bogoraz, 1909).

According to some authors, the shamanistic practices of indigenous Siberian tribes prominently featured consuming fly agaric mushrooms to produce states of altered consciousness, occasionally in combination with either bog bilberry juice (Lewin, 1924) or with the alcoholic drinks made from it (Ohlmarks, 1939). These sources however do not mention that the bog bilberry would have any intoxicating effect by itself, aside from the alcohol content. Later citations, including Encyclopedia of Psychoactive Plants (Rätsch, 2005), make this assumption by themselves.

There exist several reports from the Eastern front of World War II, collected and published in 1941 and 1944 in the German journal “Sammlung von Vergiftungsfällen” (“A collection of poisoning cases”). The first, written by the army surgeon L. Lendle, reports on a troop of soldiers stationed near Smolensk who often picked and ate “blueberries” that reportedly had white pulp, as opposed to common bilberries. After the ingestion of one such forage, following symptoms appeared in one of the soldiers: intoxication, vomiting, dilated pupils, redness in the face and dryness of the throat. These symptoms resolved itself before the affected soldier could be transported into the field hospital and the soldier failed to bring a sample of the ingested berries with him. Based on these symptoms, Lendle first postulated that it was a simple case of a deadly nightshade (*Atropa belladonna* L.) poisoning. However, upon questioning, the soldier claimed that there was no nightshade growing in the area and from his description, the berry most likely ingested was that of *Vaccinium oxycoccos* L., a cranberry^2^. Lendle briefly entertains a possibility of accidental ingestion of a bog bilberry, which was known to him as potentially poisonous, but he dismisses this in the end. In his report, he asks for a closer inspection of the cases of berry poisoning in Russia, which were known to happen in the World War I as well (Lendle, 1943).

The second collection comes from the pharmacologist K. Zipf, who reports more similar cases from the Eastern front and in two cases he was able to receive a sample of the ingested berries. In one case, which happened south of Lake Peipus, a soldier experienced bradycardia, dilated pupils and tingling in his arms after ingesting “bilberries”. In the second case, the affected soldier ate “blueberries” and complained of dizziness, flickering in the eyes and a strong feeling of weakness, which worsened in the next hours. At one point he could only lie on his back with his legs raised and his heart rate was 32/min. On the next day, he felt well again. A number of similar reports was observed in the same troop in the course of summer 1941. In both cases, the provided plant sample was identified as *V. uliginosum*. Based on the fact that bradycardia was present in all cases, extracts of the berries were tested for parasympathomimetic effects, with no observable changes in all models and samples. In the end of his report, Zipf suggests that the parasitic fungus *Monilinia megalospora* (Woronin) Whetzel might be responsible for these effects (Zipf, 1944).

### 3.8 China, Mongolia, Japan

To the best of the authors‘ knowledge, there is no reference towards the potential toxicity in Chinese and Mongolian literature and culture. The berry is regarded as an excellent food source and the annual wild harvest of *V. uliginosum* in China is 7 million kilograms, which is primarily used for producing wine, juice, syrup, jam, and preserved products (Liu et al., 2010; Wang et al., 2019). It is primarily harvested from the wild stands in the northernmost part of China, where the core of *V. uliginosum* habitat is located: Greater and Lesser Khingan Mountains and Changbai Mountains (Yang et al., 2018; Li et al., 2022).

In Japan, the berries are eaten raw or processed into jams and jellies (Masuoka et al., 2007).

### 3.9 Caucasus and Turkey

In this region, *V. uliginosum* occurrs rarely, in the high elevations of Caucasus and northeast Anatolia. It is occasionally picked and eaten and regarded as edible and nutritious (Rikli, 1913; Akbulut, 2022; Harutyunyan et al., 2023). Leaves of bog bilberry were used as a tea substitute called Batum-tea in Georgia (Lewin, 1924).

## 4 Search for the toxic principle in *V. uliginosum* berries

The search for the toxic principle of the berries of *V. uliginosum* was occasionally attempted throughout the 20th century, but without any conclusive results.

The first chemical analysis was attempted by Feder in 1912. His comparative analysis of bog bilberries and common bilberries was unfortunately limited only to botanical descriptions and a brief chemical analysis of sugars, acids, nitrogen, alkalinity and mineral content of the berry juice (Feder, 1912).

The first toxicological testing of bog bilberries was carried out in Poland at the State Institute for Food Testing in Lodz following the incident involving Henryka Frenkiel’s son. The results showed that the berry sample contained a substance which had a hemolytic effect on defibrinated animal blood. On further testing, this substance proved to be a saponin-like compound. This substance was, however, not responsible for the boy’s violent reaction to bog bilberries (Section 3.3, Frenkiel, 1932).

A brief toxicological testing was done at the Pharmacology Institute in Königsberg (nowadays Kaliningrad) in 1944 after the reports of poisoning at the Eastern front of WWII. Extracts of the berries, prepared according to the Stas-Otto separation method, were tested on a living cat to observe blood pressure changes, on an isolated dog pupil and an isolated dog intestine for parasympathomimetic effects, with no observable changes in all models and samples (Zipf, 1944).

The first systematic analysis of bog bilberries was carried out in Poland by Piechocka and Szymczyk in 1955. They prepared an array of extracts aimed to concentrate alkaloids and other potentially toxic glycosides; these extracts all produced negative reactions with alkaloid-specific reagents. All extracts were also tested on mice (10 mice per extract, 70 in total), none of them produced any negative effects; two recorded deaths were caused by catheter injury. Moreover, Piechocka herself ingested three 250 g servings of fresh bog bilberries every 30 min, 750 g in total; there were no noticeable effects. The authors conclude that the negative effects described by some are possibly a result of individual allergy or intolerance, such as with strawberries or raspberries (Piechocka and Szymczyk, 1955).

Hultin and Torssell performed a semi-quantitative screening of common Swedish plants for the content of alkaloids using 6 different alkaloid-specific reagents. The whole plant extract of *V. uliginosum* gave a negative reaction, as did *V. oxycoccus*, *L. palustre*, *E. nigrum* L., *Andromeda polifolia* L. and *Arctostaphylos uva-ursi* (L.) Spreng. Only *V. myrtillus* and *V. vitis-idaea* gave weak positive reactions (Hultin and Torssell, 1965).

Referencing many of the above-mentioned cases of poisoning by bog bilberries, another study proved the absence of alkaloids and toxic diterpenes in the leaves of bog bilberry from Belgium. Instead they identified several triterpenes and β-sitosterol (Nees et al., 1973). A similar study from few years later found trace amounts of alkaloids in leaves of *V. uliginosum* (the extract produced a cloudy reaction with Dragendorff’s and Wagner’s reagent) (Jung et al., 1979).

After this publication, the mentions of bog bilberry toxicity faded away from scientific literature and newly published research articles do not mention it at all. However, some more recent *in vivo* and *in vitro* experiments include relevant data, i.e., cell viability and organ toxicity, for bog bilberry fruit extracts used in these assays.

McGill (2010) reported the cell viability of serum deprived SH-SY5Y neuroblastoma cells supplemented with bog bilberry extracts (aqueous and acetone/water 70/30, in concentrations 5 and 75 μg/mL). In both concentrations, the extracts had no significant effect on the cell viability, with a trend towards increased viability (McGill, 2010). Esposito et al. (2019) reported that the 70% acidified aqueous methanolic extract of Alaskan bog bilberries had no significant effect on cell viability of primary human dermal fibroblasts (HDFa) in the range of 50–250 μg/mL (Esposito et al., 2019). Collin (2021) reports that a methanolic extract of Alaskan bog bilberries had a beneficial effect on cell viability of colorectal adenocarcinoma (Caco-2) cells at concentrations 5–10 μg∕mL, but after acute exposure to 100 μg∕mL of the extract for 4 h, the relative cell viability was reduced to 64.2% (Collin, 2021). Much higher concentrations of an anthocyanin-rich bog bilberry extract were used in a study on hepatocellular carcinoma cell line (Hep-G2) and colorectal adenocarcinoma cell line (Caco-2), up to 1.5 mg/mL. A concentration-dependent reduction of cell viability was observed, starting at 300 μg∕mL, until the cell viability reached approx. 10% at approx. 1 mg/mL. It was suggested by the authors of this study that anthocyanins may specifically reduce cell viability of cancer cells, as opposed to nonmalignant cell lines, but this hypothesis remains unproven (Liu et al., 2010).

Kim and Choung (2012) reported that there were no signs of liver and kidney toxicity observed in mice treated by oral administration of bog bilberry polyphenols and anthocyanins during the treatment of atopic dermatitis. The cohort of 40 mice was administered the extract in concentrations up to 40 μg/kg body weight (Kim and Choung, 2012). A cohort of 150 C57BL6/J mice supplemented with Alaskan bog bilberry powder (5% of total food pellet weight) significantly improved anxiety-like (open field), motor coordination (parallel rod floor test), sensory (cylinder test) and cognitive (novel object recognition) behaviors when introduced to neurotoxic challenge with MnCl_2_. There was no indication that the bog bilberry itself had any neurotoxic or behavior-altering effect on mice (Maulik, 2018)

## 5 The hypotheses for bog bilberry toxicity

**FIGURE 3:**
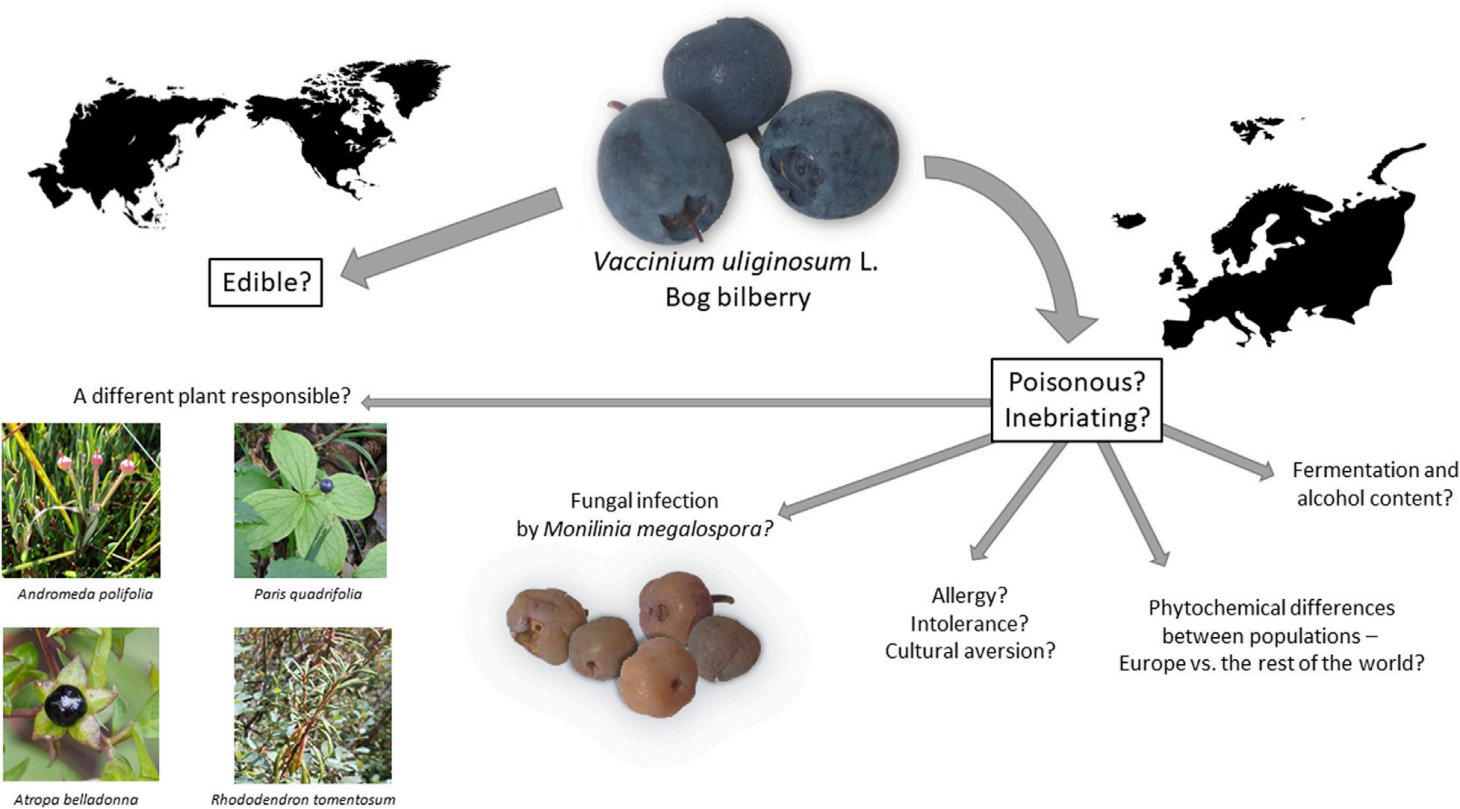
A summary of the hypotheses behind the toxicity of the bog bilberry.

The hypotheses mentioned in the literature, as well as other possible explanations for the incidences of poisoning by bog bilberry are summarized in Figure 3.

### 5.1 *Monilinia megalospora* (Woronin) Whetzel

*Monilinia* is a genus of parasitic fungus that grows on various plants of the genus Rosaceae and Ericaceae. There are around 30 described species of *Monilinia*. Those that grow on berries of the genus Ericaceae are *Monilinia Vaccinii-Corymbosi* (J.M.Reade) Honey (on cultivated blueberries), *M. oxycocci* (Woronin) Honey (on cranberry—*V*. *oxycoccos* and *V. macrocarpon*), *M. baccarum* (J.Schröt.) Whetzel (on bilberry *V. myrtillus*), *M. urnula* (Weinm.) Whetzel (on lingonberry *V. vitis-idaea*) and *M. megalospora*. They were described for the first time by the Russian biologist Mikhail Stepanovich Woronin in the 1880s. The current consensus among the mycologists is that they represent separate species and not simply an ecomorphosis of the same organism. They produce two types of spores that have different dispersal mechanisms and infect specific host tissues. Each spore type is produced only once in a growing season and has very limited opportunity to cause infection. In spring, apothecia develop on overwintered mummified fruits (termed pseudosclerotia) and release ascospores that initiate primary infection of newly emerging tissues, namely, shoots and young leaves. Asexual spores (macroconidia) are produced on blighted plant parts and subsequently spread infection to flowers by landing on the stigma. They form mycelia in developing berries, turning them into so-called “mummified berries“ (Woronin, 1888; Kotlaba and Pilát, 1952; Batra, 1983; Munda, 2011).

The theory that a *Monilinia* infection is responsible for the inebriating effects of the bog bilberry was suggested by Zipf, 1944, probably for the first time (Section 3.7), and was subsequently cited by many sources (mainly Rätsch, 2005). Although on the first sight appearing plausible (in comparison, for example, with the infection of cereal crops with ergot fungus, *Claviceps purpurea*), it leaves unanswered questions. Mainly, many *Vaccinium* species, be it cultivated or wild, can be infected by a *Monilinia* fungal disease, therefore *V. uliginosum* and *M. megalospora* would have to possess some unique characteristics among them. To prove this theory, a phytochemical and toxicological analysis of *Monilinia*-infected berries is necessary.

There have been no studies yet regarding the toxic compounds in *Monilinia* genus growing on Ericaceae berries. The only scientific attention they have gotten so far are studies analysing the morphology and life cycle of the fungus, impact of *Monilinia* blight on berry plantations, strategies to prevent them and analyses of the stress-responsive genes in *Vaccinium* plants (Woronin, 1888; Kotlaba and Pilát, 1952; Munda, 2011; Jose et al., 2021). Not even *M. Vaccinii-Corymbosi* has been investigated for potential toxicity, despite being the most severe disease of commercially grown blueberries, accounting for up to 70% crop loss in some plantations if left unmanaged (Cucak et al., 2022).

### 5.2 Accidental ingestion of a different plant

During careless berry picking, one can accidentally (or unknowingly) ingest parts of another, poisonous plant and such cases are not uncommon even today. Bog bilberry can be confused with other blue berries, such as *Lonicera nigra* L., *Paris quadrifolia* L., *A. belladonna* L. or *Solanum nigrum* L. Parts of other poisonous plants that grow in the same habitat can also be accidentally ingested, e.g., *Andromeda polifolia* L., *Rhododendron ferrugineum* L. or *Rhamnus frangula* L.

The symptoms of poisoning described by Kreuder, 1937; Zipf, 1944 are characteristic for grayanotoxin poisoning, which specifically includes low heart rate, vision disturbances, diaphragm paralysis and breathing difficulties, loss of coordination, weakness and paresthesia in the extremities and around the mouth. This is caused by opening of the sodium channels in *nervus vagus* (Aronson, 2016). Grayanotoxins are diterpenoid substances which occur in several plant species from the Ericaceae family, including *A. polifolia* and many *Rhododendron* species (Plugge, 1885), but not in the genus *Vaccinium* (Nees et al., 1973; Booth et al., 2012).

Other cases described by Lendle and Zipf point towards tropane alkaloid poisoning, with vomiting, mydriasis, dry mouth and redness of face as the telltale symptoms. This happens after ingesting poisonous berries from the nightshade family (Solanaceae) (Adamse et al., 2014).

*Rhododendron tomentosum* Harmaja (i.e., *Ledum palustre* L.) is considered responsible for the inebriating effects of bog bilberry by some. This species, also known as Labrador tea or Marsh rosemary, is a small fragrant shrub growing in circumboreal regions of the entire northern hemisphere (Canada, Greenland, Scandinavia, Baltic region and Siberia), as well as a glacial relict in bogs of some Central European countries (Gibbs et al., 2011). The plant is a source of essential oil, with the major components being (+)-ledol and (−)-palustrol. These sesquiterpenes can affect the central nervous system after high dose intake, initially leading to psychomotor stimulation, then to seizures and cramps, finally to paralysis, breathing problems and even death (Marles et al., 2000; Dampc and Luczkiewicz, 2013). However, the leaves of Labrador tea are used as a medicinal herb and taken as tea against coughs, colds and indigestion by many indigenous tribes of the circumboreal area (Russell, 1994; Garibaldi, 1999). The toxic effects are usually manifested only after ingesting very high doses, but the fragrant essential oil might be irritating to more sensitive individuals (Aronson, 2016). The theory appearing in eastern Europe claims that such sensitive individuals would brush against the bushes, releasing and breathing in the essential oil, which then irritates the respiratory system and brings upon cough, dizziness and headache. This is more probable than the Polish variation in which the *Rhododendron* pollen contaminates the berries; nevertheless, it is unlikely that the inhaled dose of volatile sesquiterpenes would be sufficient to produce hallucinations or life-threatening side effects. Moreover, *R. tomentosum* and *V. uliginosum* cannot always be found growing at the same site.

### 5.3 Intolerance, allergy, diseases

The report of Frenkiel, 1932 describes a violent reaction of her son to ingested bog bilberries, similar to favismus or black water fever. Both of these diseases are nowadays known to be caused by glucose-6-phosphate dehydrogenase enzyme deficiency. It is generally more serious in children than in adults and it is an X-chromosome-linked recessive disorder, therefore affects boys more than girls. Blueberries are a known trigger of G6PD-related hemolysis (Babu et al., 2019), therefore it can be assumed that the same mechanism was responsible for this incident of bog bilberry toxicity.

Although berries of the genus *Vaccinium* are generally considered hypoallergenic, there are known cases of blueberry allergy, which manifests with flush, itching, swelling and shortness of breath. This is in some cases caused by an immune system reaction to a 10 kDa protein contained in blueberries (Gebhardt et al., 2009; Dereci et al., 2015).

Vomiting and headaches after consuming large amounts of blueberries or bilberries are known phenomena (Jones, 2010) and can be caused by a sensitivity of the digestive tract to tannins and tannin-rich foods in some individuals, especially children (Netoliczky, 1914; Cerutti et al., 2020).

Lastly, despite it being a rare occurrence in Europe, alveolar echinococcosis is a life-threatening disease caused by a parasitic tapeworm *Echinococcus multilocularis*. The eggs are spread by animal feces and can be ingested after eating unwashed wild berries. Such cases have been reported from Germany. The general public in Germany and France has been warned against eating wild berries without thoroughly washing them. Alveolar echinococcosis is, however, characterized by an asymptomatic incubation period of 5–15 years and only afterwards manifests itself with abdominal pain, nausea and vomiting (Nunnari et al., 2012; Malkamäki et al., 2019). Therefore, while being a reason for some people to avoid wild-picked berries in general, this disease does not explain the targeted aversion towards bog bilberries.

### 5.4 Alcohol

Literature references point out that bog bilberries ferment rapidly in storage, apparently faster and better than any other wild berry gathered in the boreal and arctic regions in the Northern hemisphere (Schübeler, 1873; Jones, 2010). They were used to make alcoholic drinks in many parts of the world (Schübeler, 1873; Bogoraz, 1909; Leisner, 1940; Gajek, 1981) and this may be the reason why they are often called “intoxicating berry” or “drunk berry” (Netoliczky, 1914). This is also consistent with the fact that the case reports of bog bilberry toxicity peter out after 1950s, with the advance of modern food conservation and refrigeration technology.

The custom of Siberian tribes to mix bog bilberry alcohol with fly agaric mushrooms (Ohlmarks, 1939) may have added to the overall bad reputation of *V. uliginosum*; it is easy to assume after hearing about thusly produced states of altered consciousness, that the bog bilberry itself (“mad berry,” “intoxicating berry”) is responsible for the hallucinogenic effect and not the fly agaric constituents.

### 5.5 Phytochemical differences between different populations of bog bilberry

**FIGURE 4:**
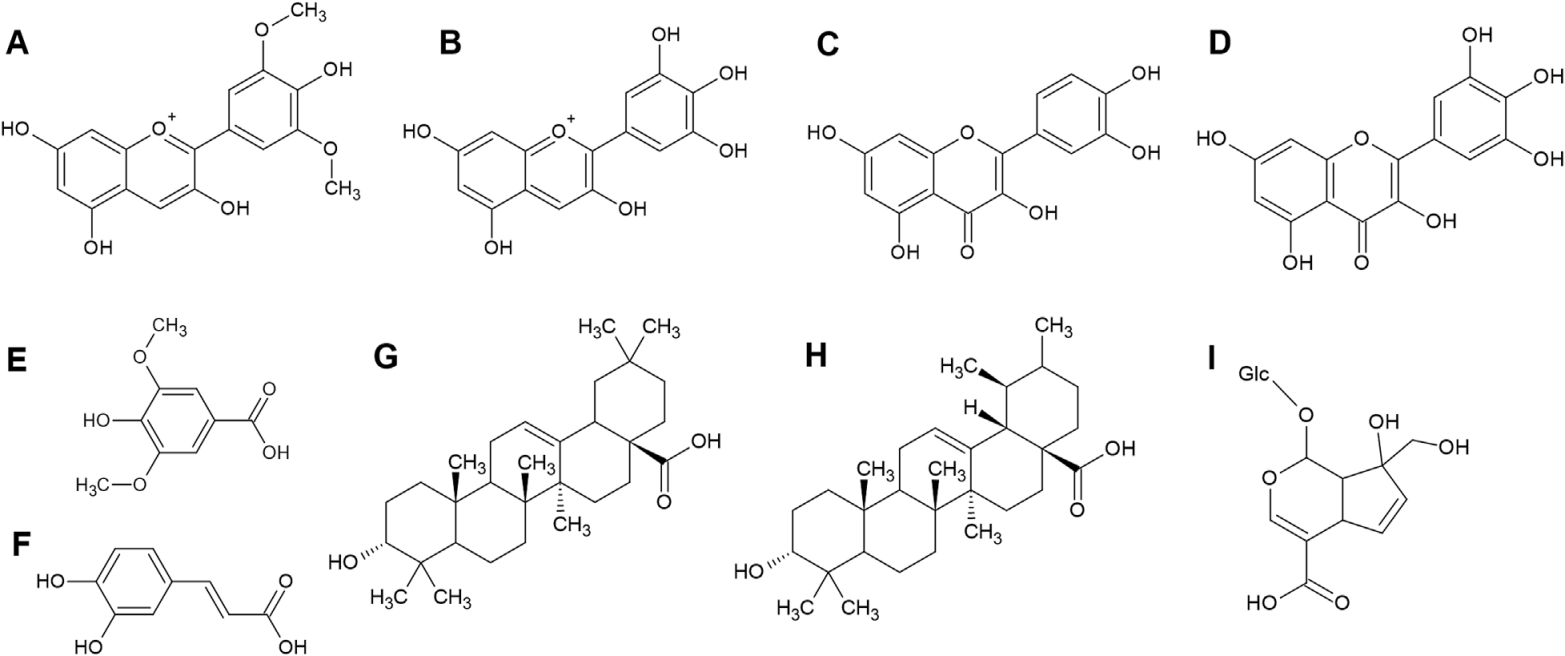
Structures of the main constituents of bog bilberry fruits: delphinidin **(A)**, malvidin **(B)**, quercetin **(C)**, myricetin **(D)**, syringic acid **(E)**, caffeic acid **(F)**, oleanolic acid **(G)**, ursolic acid **(H)** and monotropein **(I)** (Lätti et al., 2010; Ancillotti et al., 2016; Colak et al., 2016; Heffels et al., 2017).

First suggested by Kupffer in 1906, differences in phytochemical constituents might explain the different reactions of locals to bog bilberries. However, in the last 20 years multiple phytochemical analyses were performed on bog bilberry fruits from many different countries, notably Turkey (Colak et al., 2016), Armenia (Harutyunyan et al., 2023), Macedonia (Stefkov et al., 2014), Italy (Ancillotti et al., 2016), Poland (Heffels et al., 2017), Latvia (Klavins et al., 2019), Lithuania (Kraujalyte et al., 2015), Finland (Määttä-Riihinen et al., 2004; Määttä-Riihinen et al., 2005; Lätti et al., 2010; Trivedi et al., 2019), Japan (Masuoka et al., 2007), China (Hua and Yoshitama, 2006; Li et al., 2011; Wang et al., 2014; Su et al., 2016) and Alaska (McGill, 2010; Pambianchi et al., 2021). Until now, no discernibly toxic constituents were discovered and the above listed publications are in accordance with each other regarding the phytochemical make up of bog bilberry fruits (Figure 4).

The main phenolic constituents are anthocyanins cyanidin, delphinidin, malvidin, peonidin and petunidin. From this list, malvidin is the most abundant. These compounds are mainly present in the form of their glycosides such as arabinosides, galactosides, glucosides, xylosides and diglucosides (Li et al., 2011; Ancillotti et al., 2016; Colak et al., 2016).

The main flavonoid of bog bilberry fruits is quercetin, with myricetin, laricitrin, isorhamnetin, kaempferol and syringetin in the minority. Similarly to anthocyanins, they are mainly present in the glycosylated form, with glucose, galactose, arabinose and rhamnose as the predominant sugar moieties (Lätti et al., 2010; Li et al., 2011; Ancillotti et al., 2016; Lee et al., 2016). Catechin and epicatechin are also present in bog bilberries (Ancillotti et al., 2016).

Esculetin and scopoletin, belonging to the class of simple coumarins, are found in bog bilberries (Ancillotti et al., 2016). The fruits also contain iridoids, namely, deacetylasperulosidic acid, loganic acid, monotropein, scandoside and splendoside (Lee et a., 2016; Heffels et al., 2017).

In addition, a great number of organic acids can be found within bog bilberries. These include syringic acid, caffeic acid, chlorogenic acid, coumaric acid, ferulic acid, gallic acid, protocatechuic acid, quinic acid, salicylic acid, and their derivates (Ancillotti et al., 2016; Colak et al., 2016).

Two triterpenoid acids, oleanolic and ursolic acid, can be found in the wax layer covering the bog bilberry fruits (Trivedi et al., 2019).

It is important to mention that several environmental factors, including light, temperature and altitude, are known to have an impact on the metabolite profile in fruits of *V. uliginosum L.* from various geographical origins, particularly the content of anthocyanins and flavonols. (Su et al., 2016).

### 5.6 Cultural aversion

It is not uncommon that two cultures, not necessarily geographically separate, have highly divergent opinions on the edibility of a natural resource. Such is the case of tundra mushrooms: while people at the Russian side of the Bering Strait pick mushrooms with great enthusiasm, the native tribes on the Alaskan side avoid them completely; moreover, the native populace of Siberia only took up mushroom-picking after being influenced by Russian immigrants (Yamin-Pasternak, 2008).

It can be hypothesized that the aversion of European nations towards bog bilberries does not have a clear origin at all and instead stands on the intersection of a limited cross-cultural contact and a few unpleasant experiences or alarming stories (not necessarily caused by *V. uliginosum*), which then remained in the cultural memory and were passed on through generations.

## 6 Discussion

Presently, it is still unclear where the information about the bog bilberry toxicity originated; locals from European countries in which this knowledge is present agree that the berries cause unpleasant and inebriating effect upon ingestion, but differ in the exact description of the effect and what causes it. This is then in strong contrast with other parts of the world where no such tales exist and the berries are freely eaten by the local people.

Some case reports, such as that provided by Frenkiel in 1932, might now be explained by using modern knowledge of pathobiochemistry, where bog bilberry was indeed the causative agent of the described disorders. Other incidences, such as those reported by Kreuder 1937, Lendle 1943; Zipf 1944, still cannot be fully explained, although an accidental ingestion of a different poisonous plant may be plausible. This is further supported by the fact that whenever a toxicity testing was attempted under controlled conditions, no harmful effects were observed *in vitro* and *in vivo*.

Judging from the recent phytochemical investigations of bog bilberry fruits it appears there are no toxic constituents present in them. Nevertheless, the aversion towards bog bilberries present to this day substantiates further investigations in order to unambiguously confirm the edibility of this berry. Upon confirmation, bog bilberry could prove a worthy source of food and bioactive compounds, especially in Europe where it is still underutilized or completely avoided. Bog bilberry is hardy and has already attracted the attention of horticulture science as a potential berry crop in particularly harsh conditions of Arctic tundra (Holloway, 2006).

The directions in the future toxicological research that need to be explored include chemical and toxicological analyses of *Monilinia*-infected berries, comparison between different habitats and an assay-targeted search for potentially toxic constituents of the bog bilberries. Other hypotheses, such as the contamination with *Rhododenron tomentosum* or changes in phytochemistry upon fermentation, are likewise worth exploring.

## Footnotes

1 The species mentioned here is confusing; What we nowadays know as *Lonicera xylosteum* bears poisonous bright red berries; search for the name *Hundslilie* does not return any applicable hits; the plant known as *Specklilie* is either *Lonicera caprifolium* bearing poisonous orange berries, or *Lonicera periclymenum* bearing poisonous red berries; *Lonicera caerulea* or another variety of haskap berry with blue blueberry-like fruits are not poisonous. It might have been *Lonicera nigra* which used to be known as *Xylosteon nigrum* or *Caprifolium roseum* in the literature of early 20th century, when the taxonomy of Loniceraceae was not properly defined yet. *Lonicera nigra* fits the description in the text, grows in the area and has large bluish-black berries that are mildly poisonous.

2 The actual berry consumed by the soldier remains impossible to determine. Lendle claims that the soldier is to be trusted on the described plant, since he was a dentist and, according to him, “obviously intelligent,” but then described a cranberry which contradicts the earlier mention of a “blue berry white inside.” Lendle also described that the same troop got poisoned by foraged mushrooms on a different occasion, therefore their ability to identify plants and mushrooms appears dubious.

## References

[B1] Adamse P. van Egmond H. P. Noordam M. Y. Mulder P. P. J. De Nijs M. (2014). Tropane alkaloids in food: poisoning incidents. Qual. Assur. Saf. Crops Foods 6, 15–24. 10.3920/QAS2013.0314

[B2] Ainana L. Zagrebin I. (2014). Russia: Edible plants used by siberian Yupik eskimos of southeastern chukotka peninsula. Government Printing Office, The United States Department of the Interior.

[B3] Akbulut S. (2022). Importance of edible wild plants in world food security: the case of Turkey. Int. J. Agric. Sci. Food Technol. 8, 209–213. 10.17352/2455-815x.000165

[B4] Alsos I. G. Engelskjøn T. Gielly L. Taberlet P. Brochmann C. (2005). Impact of ice ages on circumpolar molecular diversity: insights from an ecological key species. Mol. Ecol. 14, 2739–2753. 10.1111/j.1365-294X.2005.02621.x 16029475

[B5] Ancillotti C. Ciofi L. Pucci D. Sagona E. Giordani E. Biricolti S. (2016). Polyphenolic profiles and antioxidant and antiradical activity of Italian berries from *Vaccinium myrtillus L.* and *Vaccinium uliginosum L.* subsp. gaultherioides (Bigelow) S.B. Young. Food Chem. 204, 176–184. 10.1016/j.foodchem.2016.02.106 26988491

[B6] Anderson J. P. (1939). Plants used by the eskimo of the northern bering sea and arctic regions of Alaska. Am. J. Bot. 26, 714–716. 10.1002/j.1537-2197.1939.tb09343.x

[B7] Andre A. Fehr A. (2000). Gwich’in ethnobotany. Inuvik: Gwich’in Social and Cultural Institute and Aurora Research Institute.

[B9] Aronson J. K. (2016). “Ericaceae,” in Meyler’s side effects of drugs (Germany: Elsevier), 93–96. 10.1016/b978-0-444-53717-1.00697-1

[B10] Babu T. Panachiyil G. M. Sebastian J. Ravi M. D. (2019). Probable blueberry-induced haemolysis in a G6PD deficient child: a case report. Nutr. Health 25, 303–305. 10.1177/0260106019885290 31707919

[B11] Batra L. R. (1983). Monilinia vaccinii-corymbosi (sclerotiniaceae): its biology on blueberry and comparison with related species. Mycologia 75, 131–152. 10.1080/00275514.1983.12021642

[B12] Berg R. (1913). Über die Moor-oder Rauschbeere. Pharm. Zentralhalle Dtschl 54, 452–453. 10.24355/dbbs.084-201909181427-0

[B13] Bertová L. (1982). “Flóra slovenska, 1st ed. (Bratislava: Veda, vydavateľstvo Slovenskej akadémie vied).

[B14] Bogoraz W. (1909). The chukchee. Leiden: E. J. Brill.

[B15] Booth N. L. Kruger C. L. Wallace Hayes A. Clemens R. (2012). An innovative approach to the safety evaluation of natural products: cranberry (*Vaccinium macrocarpon Aiton*) leaf aqueous extract as a case study. Food Chem. Toxicol. 50, 3150–3165. 10.1016/j.fct.2012.03.075 22504784

[B16] Borisova I. Z. Illarionov V. V. Illarionova T. V. (2018). Cultural heritage in the food traditions of the Sakha people. J. Fundam. Appl. Sci. 9, 1388. 10.4314/jfas.v9i2s.850

[B17] Bremensis A. Waitz G. Lappenberg J. M. (1876). Adami Gesta Hammaburgensis ecclesiae pontificum ex recensione Lappenbergii. 2nd ed. Hahn: Hahn, Hannoverae.

[B18] Cerutti F. Crescio M. I. Costantini A. Acutis P. L. Vaudano E. Peletto S. (2020). WINE INTOLERANCE IN ITALY: A PILOT STUDY. Ital. J. Food Sci. 32, 734–742. 10.14674/IJFS.1853

[B19] Colak N. Torun H. Gruz J. Strnad M. Hermosín-Gutiérrez I. Hayirlioglu-Ayaz S. (2016). Bog bilberry phenolics, antioxidant capacity and nutrient profile. Food Chem. 201, 339–349. 10.1016/j.foodchem.2016.01.062 26868586

[B20] Collective of Elders (2014). A guide to the ethnobotany of the yukon-kuskokwim region. Fairbanks: University of Alaska Fairbanks.

[B21] Collin A. (2021). 3T3-L1 adipocytes as a model of Glut4 translocation. Alaska: University of Alaska.

[B22] Cucak M. Harteveld D. O. C. Wasko DeVetter L. Peever T. L. Moral R. de A. Mattupalli C. (2022). Development of a decision support system for the management of mummy berry disease in northwestern Washington. Plants 11, 2043. 10.3390/plants11152043 35956520 PMC9370572

[B23] Dampc A. Luczkiewicz M. (2013). Rhododendron tomentosum (Ledum palustre). A review of traditional use based on current research. Fitoterapia 85, 130–143. 10.1016/j.fitote.2013.01.013 23352748

[B24] Dereci S. Orhan F. Koca T. Akcam M. (2015). Prevalence of blueberry allergy in a Turkish population. Ann. Allergy, Asthma Immunol. 114, 259–260. 10.1016/j.anai.2014.12.016 25744908

[B25] Esposito D. Overall J. Grace M. H. Komarnytsky S. Lila M. A. (2019). Alaskan berry extracts promote dermal wound repair through modulation of bioenergetics and integrin signaling. Front. Pharmacol. 10, 1058. 10.3389/fphar.2019.01058 31611784 PMC6776586

[B26] Feder E. (1912). Über die Heidelbeere und die Rauschbeere. Pharm. Zentralhalle Dtschl 53, 1321–1323. 10.24355/dbbs.084-201912171235-0

[B27] Fienup-Riordan A. Rearden A. Meade M. Jernigan K. (2020). Yungcautnguuq nunam qainga tamarmi = All the land’s surface is medicine: edible and medicinal plants of southwest Alaska. Fairbanks: University of Alaska Press.

[B28] Frenkiel H. (1932). Ein Fall von Hämoglobinurie bei einem Kinde nach Genuß von Rauschbeeren (*Vaccinium uliginosum*). Z Kinderheilkd 52, 608–612. 10.1007/BF02286773

[B8] Furuset K. (2016). Hva betyr plantenavna blokkebaer, mikkelsbaer og skinntryte? Blyttia - Norges Bot. Ann. 74, 200–203.

[B29] Gajek J. (1981). Polski atlas etnograficzny. 1st ed. Warszawa: Instytut Historii Kultury Materialnej Polskiej Akademii Nauk.

[B30] Garibaldi A. (1999). Medicinal flora of the Alaska natives, 1st ed. Alaska nature heritage program, Anchorage.

[B31] Gebhardt C. Vieths S. Gubesch M. Averbeck M. Simon J. C. Treudler R. (2009). 10 kDa lipid transfer protein: the main allergenic structure in a German patient with anaphylaxis to blueberry. Allergy Eur. J. Allergy Clin. Immunol. 64, 498–499. 10.1111/j.1398-9995.2008.01923.x 19220224

[B32] Gibbs D. Chamberlain D. Argent G. (2011). The red list of rhododendrons. Richmond, VA: Botanic Gardens Conservation International

[B113] Grimm J. Grimm W.(1893). Deutsches Wörterbuch, achter Band. Leipzig, Germany: Verlag von S. Hirzel.

[B33] Harfmann C. (2018). Wunderbare heidelbeeren. Moosgrüne natur. Available at: https://www.moosgruene-natur.at/heidelbeeren (Accessed October 31, 23).

[B34] Hartwich C. (1911). “Andere alkoholische Getränke. Weine aus Beeren,” in Die menschlichen genussmittel. Ihre herkunft, verbreitung, geschichte, anwendung, bestandteile und wirkung (Leipzig: Tauchnitz), 761.

[B35] Harutyunyan Z. E. Hoveyan Z. H. Zakharyan M. G. Mikayelyan M. N. Vardanian I. V. Zh Sargsyan G. (2023). “Determination of bioactive compounds in vaccinium myrtillus L. And vaccinium uliginosum L. In Armenia,” in IOP Conference Series: Earth and Environmental Science, USA, October 31, 2023 (Institute of Physics). 10.1088/1755-1315/1212/1/012030

[B36] Heffels P. Müller L. Schieber A. Weber F. (2017). Profiling of iridoid glycosides in Vaccinium species by UHPLC-MS. Food Res. Int. 100, 462–468. 10.1016/j.foodres.2016.11.018 28964369

[B37] Henslowa M. (1979). Z badan nad wiedza ludowa o roslinach Ericaceae - wrzosowate. Slavia Antiq. 26, 239–291.

[B38] Holloway P. S. (2006). Managing wild bog blueberry, lingonberry, cloudberry and crowberry stands in Alaska. Fairbanks: University of Alaska Press.

[B39] Hoskovec L. (2007). VACCINIUM ULIGINOSUM L. – brusnice vlochyně. BOTANY.cz Available at: https://botany.cz/cs/vaccinium-uliginosum/ (Accessed 10.25.23).

[B40] Hua L. L. Yoshitama K. (2006). Analysis of flavonoids in the mature fruit of *Vaccinium uliginosum L.* of China. Orient Pharm. Exp. Med. 6, 65–67. 10.3742/OPEM.2006.6.1.065

[B41] Hultin E. Torssell K. (1965). ALKALOID-SCREENING OF SWEDISH PLANTS. Phytochemistry 4, 425–433. 10.1016/s0031-9422(00)86193-2

[B42] Husemann T. Husemann A. (1862). Handbuch der Toxikologie. Berlin: Verlag von Georg Reimer.

[B43] Jacquemart A.-L. (1996). Vaccinium uliginosum L . J. Ecol. 84, 771–785. 10.2307/2261339

[B44] Jones A. (2010). Plants that we eat: nauriat nigiñaqtuat. 2nd ed. Fairbanks: University of Alaska Press.

[B45] Jose S. Abbey J. Jaakola L. Percival D. (2021). Elucidation of the molecular responses during the primary infection of wild blueberry phenotypes with Monilinia vaccinii-corymbosi under field conditions. BMC Plant Biol. 21, 493. 10.1186/s12870-021-03281-2 34706657 PMC8549177

[B46] Jung H.-J. G. Batzli G. O. Seigler D. S. (1979). Patterns in the phytochemistry of arctic plants. Biochem. Syst. Ecol. 7, 203–209. 10.1016/0305-1978(79)90051-6

[B47] Källman S. (2002). The use of wild plants by the nomadic tundra nenets in northern Siberia. Sven. Bot. Tidskr. 96, 261–270.

[B48] Kari P. R. (1995). Tanaina plantlore. An ethnobotany of the dena’ina Indians of southcentral Alaska. 4th ed.

[B49] Kim M. J. Choung S.-Y. (2012). Mixture of polyphenols and anthocyanins from *Vaccinium uliginosum* L. alleviates DNCB-induced atopic dermatitis in NC/NGA mice. Evidence-Based Complementary Altern. Med. 2012, 461989–462015. 10.1155/2012/461989 PMC3486628 23133493

[B50] Klavins L. Kviesis J. Klavins M. (2019). Surface wax composition of wild and cultivated northern berries. Agron. Res. 17, 1337–1345. 10.15159/AR.19.032

[B51] Kleis R. (1936). Eesti Entsüklopeedia. Tartu: Loodus.

[B52] Knight C. (2015). Final report for FW13-149. Sustainable agriculture research and education projects. Available at: https://projects.sare.org/sare_project/fw13-149/ (Accessed 10.27.23).

[B53] Kokassaar U. (2006). Mittejoovastad joovikad. Eesti Lodus 8.

[B54] Kotlaba F. Pilát A. (1952). Hlízenka klikvová — sclerotinia oxycocci voron. v československu. Česká Mykol. 6, 41–44.

[B55] Kraujalyte V. Venskutonis P. R. Pukalskas A. Česoniene L. Daubaras R. (2015). Antioxidant properties, phenolic composition and potentiometric sensor array evaluation of commercial and new blueberry (*Vaccinium corymbosum*) and bog blueberry (*Vaccinium uliginosum*) genotypes. Food Chem. 188, 583–590. 10.1016/j.foodchem.2015.05.031 26041234

[B56] Kreuder F. (1937). Vergiftungserscheinungen nach reichlichem Genuß von Rauschbeeren. Samml. Vergiftungsfällen 8, 33–34. 10.1007/bf02452038

[B57] Kupffer K. R. (1906). Die angebliche Giftigkeit der Blaubeeren und Krähnenbeeren. Korresp. Naturforschervereins Riga 49, 141.

[B58] Lankmayer J. (2013). Rauschbeere (Vaccinium uliginosum). MeinBezirk.at. https://www.meinbezirk.at/lungau/c-lokales/rauschbeere-vaccinium-uliginosum_a668527 (Accessed October 31, 23).

[B59] Lätti A. K. Jaakola L. Riihinen K. R. Kainulainen P. S. (2010). Anthocyanin and flavonol variation in bog bilberries (*Vaccinium uliginosum L.*) in Finland. J. Agric. Food Chem. 58, 427–433. 10.1021/jf903033m 20000402

[B60] Lee B.-L. Kang J.-H. Kim H.-M. Jeong S.-H. Jang D.-S. Jang Y.-P. (2016). Polyphenol-enriched *Vaccinium uliginosum L.* fractions reduce retinal damage induced by blue light in A2E-laden arpe19 cell cultures and mice. Nutr. Res. 36, 1402–1414. 10.1016/j.nutres.2016.11.008 27993192

[B61] Lehbert R. (1913). Über die Heidelbeere und die Rauschbeere. Pharm. Zentralhalle Dtschl 54, 71–73. 10.24355/dbbs.084-201909181427-0

[B62] Leisner T. (1940). Loodusandide rahvapärastest kasutusviisidest. Eesti Lood. 8, 31–41.

[B63] Lendle L. Wielands H. (1943). Sammlung von Vergiftungsfällen: unter mitwirkung der Deutschen pharmakologischen gesellschaft. Samml. Vergiftungsfällen 12, 105–106. 10.1007/978-3-662-32716-6

[B64] Lewin L. (1897). Lehrbuch der Toxikologie. 2nd ed. Wien & Leipzig: Urban & Schwarzenberg.

[B65] Lewin L. (1924). “Der fliegenpilz,” in Phantastica. Die betäubenden und erregenden genussmittel. Für ärzte und nichtärzte (Berlin: Verlag von Georg Stilke), 118–124.

[B66] Li Q. Qi Y. Wang Q. Wang D. (2022). Prediction of the potential distribution of *Vaccinium uliginosum* in China based on the maxent niche model. Horticulturae 8, 1202. 10.3390/horticulturae8121202

[B67] Li R. Wang P. Guo Q. Q. Wang Z. Y. (2011). Anthocyanin composition and content of the *Vaccinium uliginosum* berry. Food Chem. 125, 116–120. 10.1016/j.foodchem.2010.08.046

[B68] Linné C. von (1772). Vollständiges Pflanzensystem nach der 13. latein. Ausgabe und nach Anleitung des holländischen Houttuynischen Werkes übersetzt. Nürnberg: Raspe.

[B69] Liu J. Zhang W. Jing H. Popovich D. G. (2010). Bog bilberry (*Vaccinium uliginosum* l.) extract reduces cultured hep-g2, caco-2, and 3t3-l1 cell viability, affects cell cycle progression, and has variable effects on membrane permeability. J. Food Sci. 75, H103–H107. 10.1111/j.1750-3841.2010.01546.x 20492295

[B70] Määttä-Riihinen K. R. Kähkönen M. P. Törrönen A. R. Heinonen I. M. (2005). Catechins and procyanidins in berries of Vaccinium species and their antioxidant activity. J. Agric. Food Chem. 53, 8485–8491. 10.1021/jf050408l 16248542

[B71] Määttä-Riihinen K. R. Kamal-Eldin A. Mattila P. H. González-Paramás A. M. Törrönen R. (2004). Distribution and contents of phenolic compounds in eighteen scandinavian berry species. J. Agric. Food Chem. 52, 4477–4486. 10.1021/jf049595y 15237955

[B72] Malkamäki S. Näreaho A. Oksanen A. Sukura A. (2019). Berries as a potential transmission vehicle for taeniid eggs. Parasitol. Int. 70, 58–63. 10.1016/j.parint.2019.01.008 30711641

[B73] Marles R. J. Clavelle C. Monteleone L. Tays N. Burns D. (2000). Aboriginal plant use in Canada’s northwest boreal forest. Vancouver: UBC Press.

[B74] Masuoka C. Yokoi K. Komatsu H. Kinjo J. Nohara T. Ono M. (2007). Two novel antioxidant ortho-benzoyloxyphenyl acetic acid derivatives from the fruit of *Vaccinium uliginosum* . Food Sci. Technol. Res. 13, 215–220. 10.3136/fstr.13.215

[B75] Maulik M. (2018). Role of dietary fat and supplementation in modulating neurodegenerative pathology in two animal model systems. Alaska: University of Alaska. [Dissertation].

[B76] McGill C. (2010). Biologically relevant secondary metabolites of *Vaccinium uliginosum*: bioassay-directed natural products identification of anti-neuroinflammatory agents in the Alaska bog blueberry. Alaska: University of Alaska. [Dissertation]. [Fairbanks (AK)].

[B77] Moeschlin S. (1964). Klinik und Therapie der Vergiftungen. 4th ed. Stuttgart: Georg Thieme Verlag.

[B78] Munda A. (2011). Monilinia pathogens of cultivated and native Vaccinium species in Slovenia. Acta Agric. Slov. 97, 99–104. 10.2478/v10014-011-0005-9

[B79] Natura (2019). Borówka bagienna – roślina trująca czy jadalna. Available at: http://zachwyconanatura.pl/borowka-bagienna-roslina-trujaca-jadalna/ (Accessed 10.27.23).

[B80] Nees H. Pachaly P. Zymalkowski F. (1973). Chemotaxonomy of Ericaceae: isolation and identification of triterpenes and steroids from *Vaccinium uliginosum* (author's transl). Planta Med. 24, 320–328. 10.1055/s-0028-1099505 4789554

[B81] Netoliczky F. (1914). Die Giftigkeit der “Rauschbeeren” (*Vaccinium uliginosum)* - ein Mißverständnis. Österreichische bot. Z. 64, 43–45. 10.1007/bf01644281

[B82] Nevinny J. (1908). Die Rauschbeere (*Vaccinium uliginosum L.*), ihre Verwechselung mit der Heidelbeere (*Vaccinium myrtillus L.*) und ihr Nachweis in den Fäces. Z Hyg. Infekt. 59, 95–121.

[B83] Nunnari G. Pinzone M. R. Gruttadauria S. Celesia B. M. Madeddu G. Malaguarnera G. (2012). Hepatic echinococcosis: clinical and therapeutic aspects. World J. Gastroenterol. 18, 1448–1458. 10.3748/wjg.v18.i13.1448 22509076 PMC3319940

[B84] Ohlmarks Å. (1939). Studien zum Problem des Schamanismus. Lund: C. W. K. Gleerup.

[B85] Pambianchi E. Hagenberg Z. Pecorelli A. Grace M. Therrien J.-P. Lila M. A. (2021). Alaskan bog blueberry (*Vaccinium uliginosum*) extract as an innovative topical approach to prevent UV-induced skin damage. Cosmetics 112. 10.3390/cosmetics

[B86] Piechocka J. Szymczyk F. (1955). Badania nad *Vaccinium uliginosum L.* Borowka bagienna. Rocz. Panstw Zakl. Hig. 6, 109–117.

[B87] Plugge P. C. (1885). Vorkommen von Andromedotoxin in verschiedenen ericaceen. Arch. Pharm. 23, 905–917. 10.1002/ardp.18852232302

[B88] Plus Jeden Deň (2016). Nástraha pri cestách. Available at: https://www1.pluska.sk/rady-a-tipy/nastraha-pri-cestach-namiesto-ovocia-vam-predaju-halucinogeny (Accessed 10.25.23).

[B89] Rätsch C. (2005). The Encyclopedia of psychoactive plants: ethnopharmacology and its applications. 1st ed. New York: Simon & Schuster.

[B90] Regele D. Grünebach M. Erschbamer B. Schönswetter P. (2017). Do ploidy level, morphology, habitat and genetic relationships in Alpine *Vaccinium uliginosum* allow for the discrimination of two entities? Preslia 89, 291–308. 10.23855/preslia.2017.291

[B91] Rikli M. (1913). Beiträge zur Pflanzengeographie und Florengeschichte der Kaukasusländer und Hocharmeniens. Naturwissenschaften 1, 993–998. 10.1007/bf01490940

[B92] Russell P. N. (1994). Ninilchik plantlore: an ethnobotany of the ninilchik dena’ina, Aleut, and Russian people, ninilchik traditional council.

[B114] sátni.org (2023). Available at: www.sátni.org (Accessed October 27, 2023).

[B93] Schübeler F. C. (1873). “Ericaceae,” in Die pflanzenwelt nordwegens: ein beitrag zur natur und culturgeschichte nord-europas (Christiania: IEEE), 276.

[B94] Shin N. Kotani A. Maruya Y. Gavrilyeva T. (2022). Can Yandex statistics and google trends be used to detect people’s interests in berries in the Russian Far East? Polar Sci. 33, 100871. 10.1016/j.polar.2022.100871

[B95] Söderwall K. F. (1884). Ordbok öfver svenska medeltids-språket. Lund: Berlingska boktryckeri- och stilgjuteri-aktiebolaget.

[B96] Stefkov G. Hristovski S. Petreska Stanoeva J. Stefova M. Melovski L. Kulevanova S. (2014). Resource assessment and economic potential of bilberries (*Vaccinium myrtillus* and *Vaccinium uliginosum*) on Osogovo Mtn., R. Macedonia. Ind. Crops Prod. 61, 145–150. 10.1016/j.indcrop.2014.06.053

[B97] Štorch E. (1918). Lovci mamutů. 1st ed. Praha: Dědictví Komenského.

[B98] Su S. Wang L. J. Feng C. Y. Liu Y. Li C. H. Du H. (2016). Fingerprints of anthocyanins and flavonols of *Vaccinium uliginosum* berries from different geographical origins in the Greater Khingan Mountains and their antioxidant capacities. Food control. 64, 218–225. 10.1016/j.foodcont.2016.01.006

[B99] Trivedi P. Karppinen K. Klavins L. Kviesis J. Sundqvist P. Nguyen N. (2019). Compositional and morphological analyses of wax in northern wild berry species. Food Chem. 295, 441–448. 10.1016/j.foodchem.2019.05.134 31174780

[B100] Turner N. J. (1997). Food plants of interior first peoples. Vancouver: UBC Press, Royal British Columbia Museum, First Peoples’ Cultural Foundation.

[B101] Vander Kloet S. P. (2009). Vaccinium. Flora of North America north of Mexico. Available at: http://www.efloras.org/florataxon.aspx?flora_id=1&taxon_id=134285 (Accessed January 16, 24).8.

[B102] Vaneková Z. Rollinger J. M. (2022). Bilberries: curative and miraculous – a review on bioactive constituents and clinical research. Front. Pharmacol. 13, 909914. 10.3389/fphar.2022.909914 35847049 PMC9277355

[B103] Vuijk D. 2023. FLORA of Iceland elements: *Vaccinium uliginosum*, northern bilberry or bog bilberry, bláberjalyng. Natural history of Iceland site. Available at: http://www.iceland-nh.net/plants/data/Vaccinium-uliginosum/vaccinium_uliginosum.html (Accessed 10.27.23).

[B104] Walker M. (1984). Harvesting the northern wild. Yellowknife: Outcrop Ltd.

[B105] Wang L. J. Su S. Wu J. Du H. Li S. S. Huo J. W. (2014). Variation of anthocyanins and flavonols in *Vaccinium uliginosum* berry in Lesser Khingan Mountains and its antioxidant activity. Food Chem. 160, 357–364. 10.1016/j.foodchem.2014.03.081 24799249

[B106] Wang Y. Yang H. Zhong S. Liu X. Li T. Zong C. (2019). Variations in sugar and organic acid content of fruit harvested from different *Vaccinium uliginosum* populations in the Changbai mountains of China. J. Am. Soc. Hortic. Sci. 144, 420–428. 10.21273/JASHS04740-19

[B107] Whitney C. W. Gebauer J. Anderson M. (2012). A survey of wild collection and cultivation of indigenous species in Iceland. Hum. Ecol. 40, 781–787. 10.1007/s10745-012-9517-0

[B108] Woronin M. (1888). Über die Sclerotienkrankheit der Vaccinieen-Beeren. Mem. Acad. Imp. Sci. St. Petersbg. Ser. VII 36, 1–49.

[B109] Yamin-Pasternak S. (2008). From disgust to desire: changing attitudes toward beringian mushrooms. Econ. Bot. 62, 214–222. 10.1007/s12231-008-9020-0

[B110] Yang Y. Cui B. Tan Z. Song B. Cao H. Zong C. (2018). RNA sequencing and anthocyanin synthesis-related genes expression analyses in white-fruited *Vaccinium uliginosum* . BMC Genomics 19, 930. 10.1186/s12864-018-5351-0 30545307 PMC6293651

[B111] Ziegler A. Joamie A. Hainnu R. (2018). Edible and medicinal arctic plants: an inuit elder’s perspective. 2nd ed. Toronto: Inhabit Media Inc.

[B112] Zipf K. (1944). Vergiftungen durch Rauschbeeren. Samml. Vergiftungsfällen 13, 139–140. 10.1007/BF02458204

